# The Effects of Irreversible Electroporation on the Achilles Tendon: An Experimental Study in a Rabbit Model

**DOI:** 10.1371/journal.pone.0131404

**Published:** 2015-06-26

**Authors:** Yue Song, Jingjing Zheng, Mingwei Yan, Weidong Ding, Kui Xu, Qingyu Fan, Zhao Li

**Affiliations:** 1 Orthopedics Oncology Institute of Chinese PLA and Department of Orthopedics, Tangdu Hospital, Fourth Military Medical University, Xi’an, Shaanxi, P.R. China; 2 Department of General Surgery, NO. 202 Hospital of PLA, Shenyang, Liaoning, P.R. China; 3 Department of Neurobiology, Fourth Military Medical University, Xi’an, Shaanxi, P.R. China; 4 Department of Electrical Engineering, Xi’an Jiaotong University, Xi’an, Shaanxi, P.R. China; Mayo Clinic Minnesota, UNITED STATES

## Abstract

**Background:**

To evaluate the potential effects of irreversible electroporation ablation on the Achilles tendon in a rabbit model and to compare the histopathological and biomechanical changes between specimens following electroporation ablation and radiofrequency ablation.

**Methods:**

A total of 140 six-month-old male New Zealand rabbits were used. The animals were randomly divided into two groups, 70 in the radiofrequency ablation group and 70 in the electroporation group. In situ ablations were applied directly to the Achilles tendons of rabbits using typical electroporation (1800 V/cm, 90 pulses) and radiofrequency ablation (power control mode) protocols. Histopathological and biomechanical evaluations were performed to examine the effects of electroporation ablation and radiofrequency ablation over time.

**Results:**

Both electroporation and radiofrequency ablation produced complete cell ablation in the target region. Thermal damage resulted in tendon rupture 3 days post radiofrequency ablation. In contrast, electroporation-ablated Achilles tendons preserved their biomechanical properties and showed no detectable rupture at this time point. The electroporation-ablated tendons exhibited signs of recovery, including tenoblast regeneration and angiogenesis within 2 weeks, and the restoration of their integral structure was evident within 12 weeks.

**Conclusions:**

When applying electroporation to ablate solid tumors, major advantage could be that collateral damage to adjacent tendons or ligaments is minimized due to the unique ability of electroporation ablation to target the cell membrane. This advantage could have a significant impact on the field of tumor ablation near vital tendons or ligaments.

## Introduction

Tendons and ligaments mechanically transmit muscle forces to bones, permitting locomotion and enhancing joint stability. However, if a critical tendon/ligament is adjacent to a tumor, the radical surgery performed to remove the tumor must frequently sacrifice nearby tendons or ligaments. For example, the resection of an aggressive or malignant tumor of the proximal fibula requires performing an en bloc extra-articular resection of the proximal fibula, as well as the lateral collateral ligament and the biceps femoris tendon (as these structures attach to the proximal fibula), leading to varying degrees of knee instability [1, 2]. After undergoing a resection of a tumor of the proximal tibia, the knee loses the quadriceps mechanism. Therefore, to better preserve joint function and the quality of life for patients after surgery, reconstructing the absent or injured tendons/ligaments is essential. Performing a complete repair of the joint capsule and reconstructing the surrounding muscle can restore joint stability and avoid dislocation [3]. Due to the limited self-restoration ability and the poor blood supply of tendons and ligaments, reconstructing a tendon/ligament is always time consuming and the final result is often unsatisfactory [4–6].

Consequently, an efficient procedure that can effectively inactivate tumor cells without compromising the biomechanical properties of tendons and ligaments is urgently needed. Irreversible electroporation (IRE) ablation is an advanced new technology that has been tested in preclinical studies to inactivate normal and tumor cells while sparing the tissue scaffold [7]. IRE induces cell death by creating permanent nanopores in the cellular membrane via the application of short intervals of high-voltage direct electrical current [8]. The IRE treatment uses multiple ultra-short pulses, which increases therapeutic effects while avoiding thermal effects [9]. Lee, E.W. et al demonstrated that IRE does not destroy connective tissue or denature collagen during hepatic ablation [10, 11]. Therefore, we hypothesize that tendons will maintain their biomechanical properties after IRE, and we have designed experiments to study the effects of IRE on tendons and the subsequent healing process.

## Materials and Methods

### Animals and Ethics Statement

A total of 140 six-month-old male New Zealand rabbits with an average weight of 2.6 ± 0.5 kg were used. The animals were randomly divided into two groups, 70 in the radiofrequency ablation (RFA) group and 70 in the IRE group. The animals in each group were then further randomly divided into three groups, 16 for histopathological assessments, 14 for microvascular perfusions and 40 for biomechanical tests. For an individual animal in biomechanical test, the left AT was chosen as a sham ablation control, and the right AT was used for ablation; while for an animal in histopathology assessment and microvascular perfusion, bilateral ATs were used for ablation. All experimental procedures involving animals were performed under a protocol that was reviewed and approved by the Ethics Committee of Animal Experiments of Tangdu Hospital, Fourth Military Medical University (permit number: TDLL2012034). All surgery was performed under anesthesia, and all efforts were made to minimize the suffering of the animals.

### Surgical Procedures and Tissue Ablation

**Fig 1:**
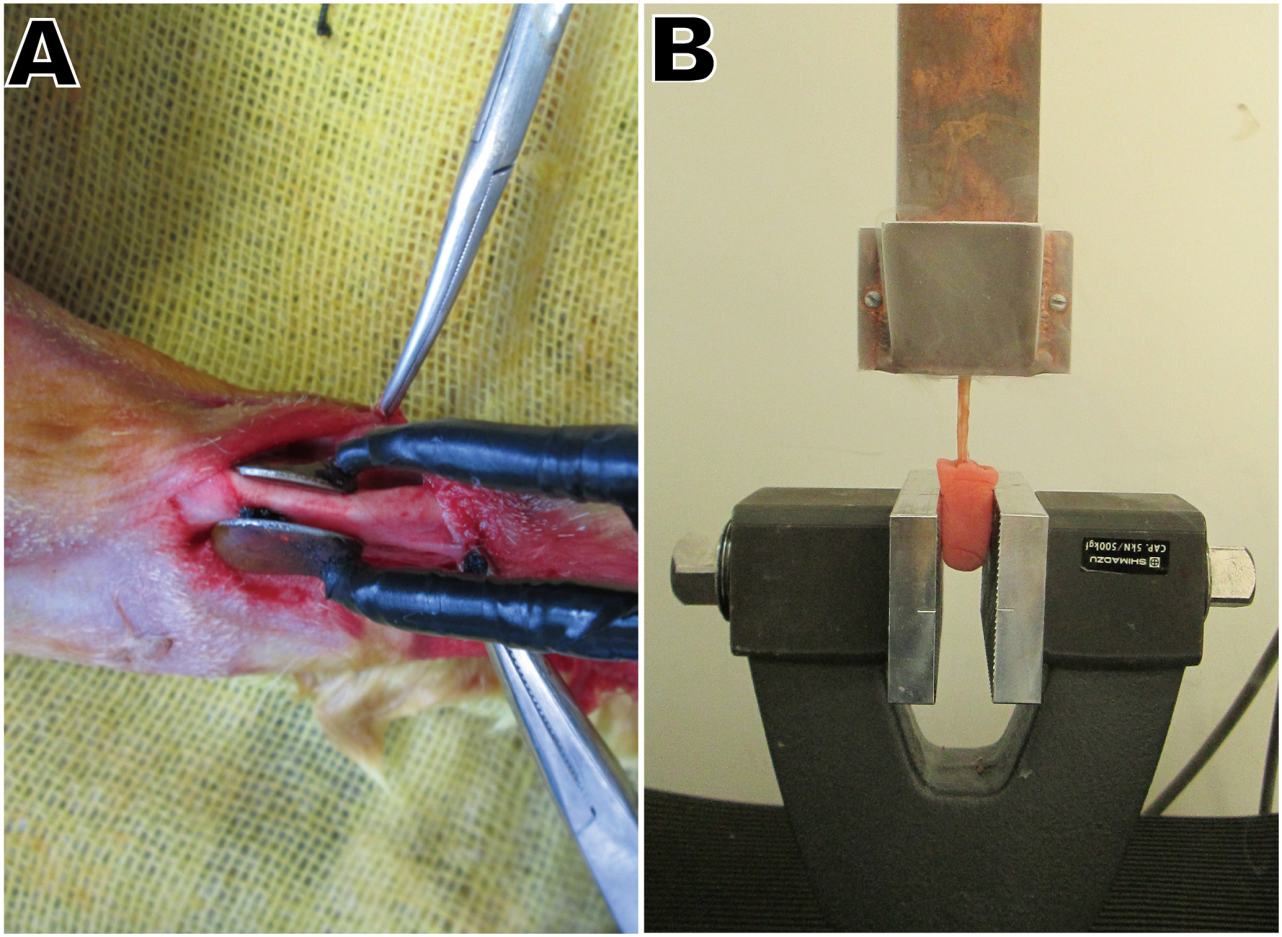
Surgical procedures and biomechanical tests. (A) Tendon exposure and preparation of the plate electrodes for ablation. (B) The apparatus used to perform the tendon biomechanical tests, including the cryo-jaw, specimens and mechanical testing clamp.

The animals were anesthetized with an intramuscular injection of diazepam (300 μg/kg) and xylazine (5 mg/kg). A segment of tendon at least 20 mm long was prepared for IRE and RFA. A hand-held clamp with two parallel metal electrodes (Tweezertrodes, 45–011, BTX, U.S.) was applied directly to the AT. The distance between the electrodes was measured with a caliper and set at approximately 3.0 mm (Fig 1A). A sequence consisting of 90, 100-microsecond long, direct current square pulses at 1800 V/cm with a pulse interval of 100 milliseconds was applied between the electrodes using an electroporation pulse generator (TP3032, Teslaman, Dalian, China). The power of the RFA was administered using RFA medical equipment (LDRF-120S, Mianyang, China). The initial RFA power was 30 W, and it increased by 10 W every 20 seconds until a sudden and major rise in impedance occurred. Three cycles were performed. The treated length was marked by two knots sutured on the peritendon. During the first 24 hours after surgery, the animals were given two doses of buprenorphine (0.05 mg/kg) and meloxicam (2 mg/kg) at eight-hour intervals. The animals were checked daily to ensure that they recovered, remained healthy, and were not experiencing pain. All tendons were harvested within the first 5 days (d) post-RFA due to AT rupture. Specimens were collected at 3 d, 1 week (w), 2 w, 4 w, 6 w, 12 w and 24 w following IRE.

### Modeling the Electrical Field

The distribution of the electrical field across the target tendon was simulated using computer software (COMSOL Multiphysics, U.S.). For the calculations, conductivity values of 4.0e^6^ [S/m], 1e^-17^ [S/m] and 0.56 [S/m] and dielectric constants of 1, 4.5 and 1050 were used for the electrodes, the insulating layer and the tendon, respectively. The conductivity and dielectric constant of the AT were measured using a signal analyzer (N9030A PXA, Agilent, U.S.).

### Histological Examinations

Specimens were prepared for histology and sections were examined and quantified by an independent observer who was blinded to treatments. Hematoxylin & eosin (H&E) staining was performed to evaluate tissue morphology. The cells on the endothelial surface of the blood vessels were stained with a mouse monoclonal CD31/PECAM1 antibody (1:50, NB600-562, Novus, U.S.), and the binding was quantified using the integral optical density (IOD) of positive staining. The number of labeled cells in the treated area was determined by randomly choosing five representative regions of interest (each with a 0.06-mm^2^ area) and counting the number of positive cells in each region using Image-Pro Plus software (version 6.0). The revascularization was defined according to the IOD value within 1 mm^2^. For transmission electron microscopy, 1×1×2-mm treated samples from each specimen were examined in longitudinal sections using a transmission electron microscope (TEM) (H-9500, Hitachi, Japan).

### Microvascular Perfusions and Microvessel Quantifications

After the animals were anesthetized, the abdominal aorta was cannulated and the vasculature of the lower extremity was perfused with 500 ml heparinized physiological saline solution (12500 IU/L). Following euthanasia, 50 ml blue polymerizing contrast compound (Microfil; Flow Tech Inc., U.S.) was injected under physiological pressure [12, 13]. Four muscle-tendon complexes from two rabbits at each time point were each divided into three segments according to the suture markers: an IRE-ablated segment, a distal segment and a proximal segment. All segments were embedded in paraffin and cut to 6-μm-thick transverse sections. Eosin staining was used to distinguish tendon tissue from blood vessels. Microvascular quantification was defined as the ratio of the number of vessel (Microfil) pixels to the total number of tendon pixels.

### Biomechanical Testing

Ten muscle-tendon complexes, each approximately 10 cm in length, were obtained at each time point. To prevent slippage of the complex from the clamps, the posterior bundle of the AT was cut. The distal segment of each complex was embedded in polymethylmethacrylate for fixation on the mechanical testing machine (SPL-10 kN, Shimadzu, Japan). We modified a simple but secure non-compressive clamping cryo-jaw to prevent the proximal segment from slipping out of the clamp [14]. The cryo-jaw features two reservoirs on each side of the copper plate and is designed to fix the gastrocnemius muscle and the end of the AT (Fig 1B). The cross-sectional area of each specimen was measured via the contact method using a micrometer (0–25 mm, Shangliang, China) with a 0.5-N load applied to the tendon. Afterwards, both reservoirs of the cryo-jaw were filled with liquid nitrogen, and the experiment began as soon as the freezing zone expanded to the border of the metal jaw (but not into the tendon). The gripping clamps were moved apart at a constant speed of 100 mm/second. No slippage out of the jaw or clamp was registered.

### Statistical Analysis

Results are expressed as the mean ± the standard deviation (SD). Two kinds of statistical methods were used. In the histological examinations and microvessel quantifications, one-way ANOVA were chosen; while in the biomechanical tests, two-way ANOVA was chosen. A Bonferroni test was used for multiple comparisons. *P* < 0.05 was taken as significant. All statistical analyses were performed using statistical analysis software (SPSS, version 17.0, Chicago, IL).

## Results

### Distribution of the Electric Field Intensity

**Fig 2:**
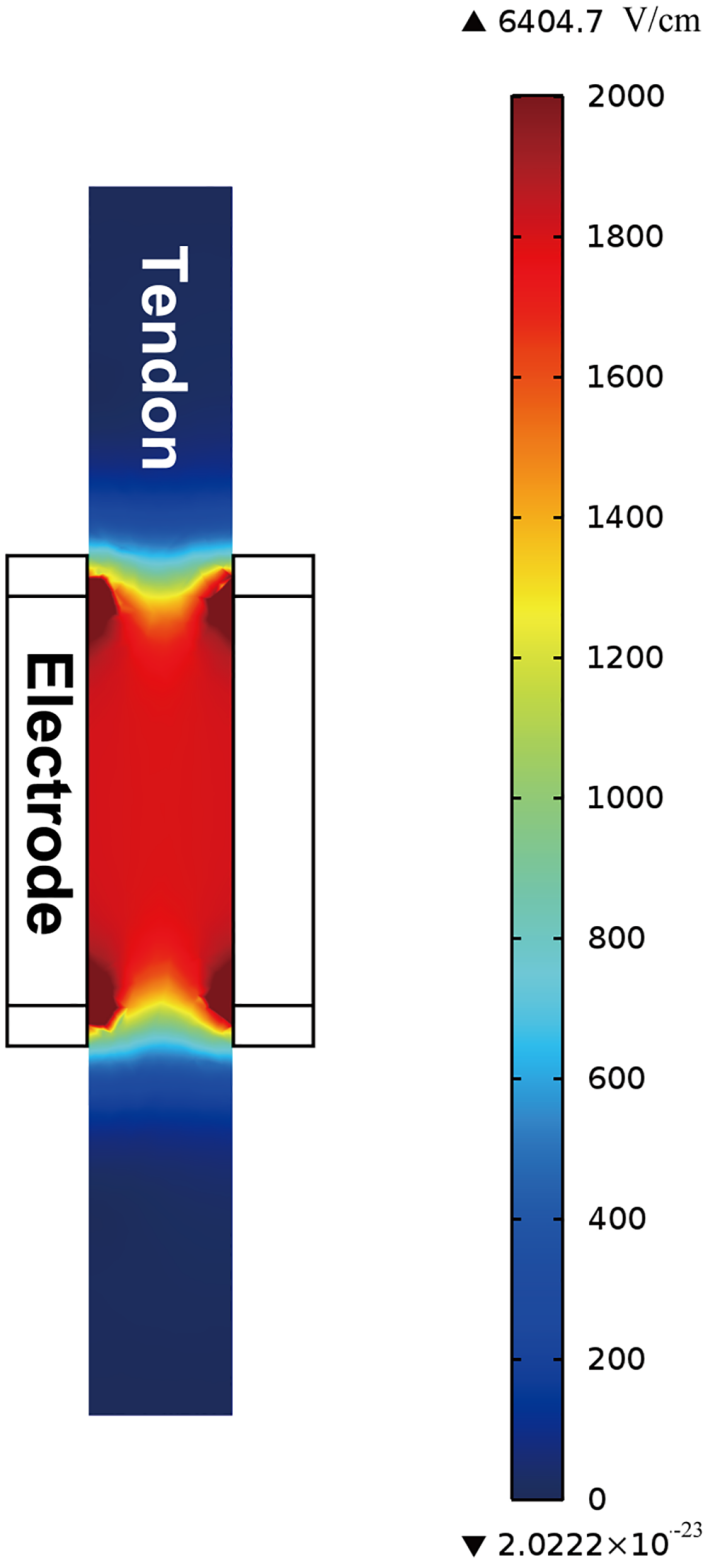
Modeling the electrical field of IRE for the AT. At 1800 V/cm, the electric field distribution between the two electrodes was nearly uniform. However, a higher electric field intensity emerged at the edge of the electrode.

Under a stimulus of 1800 V/cm, the electric field distribution between the two electrodes was nearly uniform, except for a higher electric field intensity at the edge of the electrode (Fig 2). We therefore theoretically confirmed that the electric field intensities between the two electrode plates were effectively identical.

### Clinical and Gross Pathology Observations

**Fig 3:**
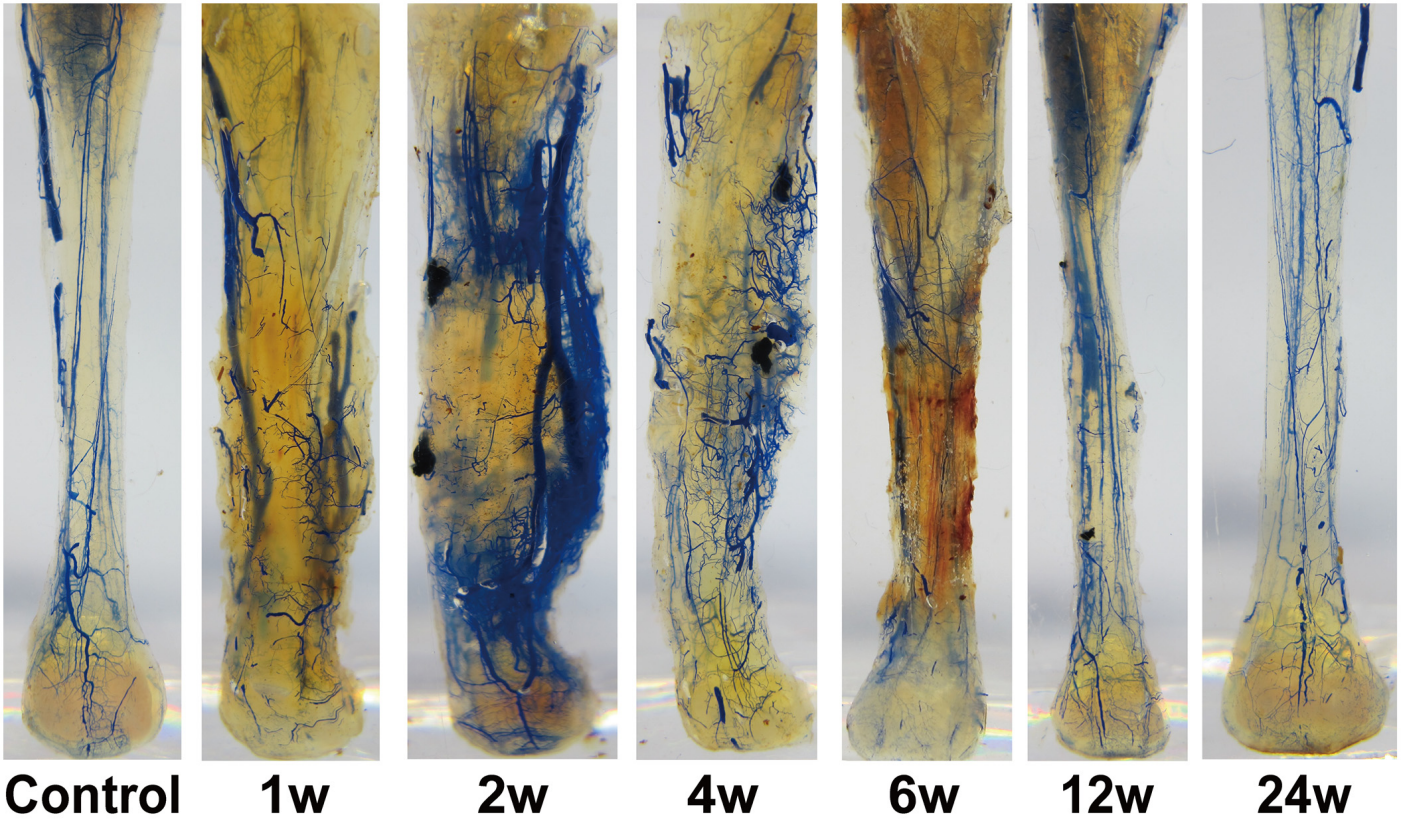
Gross specimens of IRE-ablated AT with vascular perfusion at each time point. The blue regions represent the Microfil filling in the blood vessels.

In the RFA group, AT rupture occurred at the treated zone approximately 3 d after ablation, on average. The remnants exhibited a gray, jelly-like appearance with blurred ablation boundaries. In addition, it was difficult to distinguish collagen bundles due to widespread coagulation necrosis and the subsequent liquefaction of collagens. In the IRE group, all of the animals were able to move freely, and no detectable ruptures were observed at the time of euthanasia. Compared to controls, hypertrophy of the IRE-ablated region was observed in the specimens from 3 d to 12 w following ablation (Fig 3).

### Histopathological Assessments

**Fig 4:**
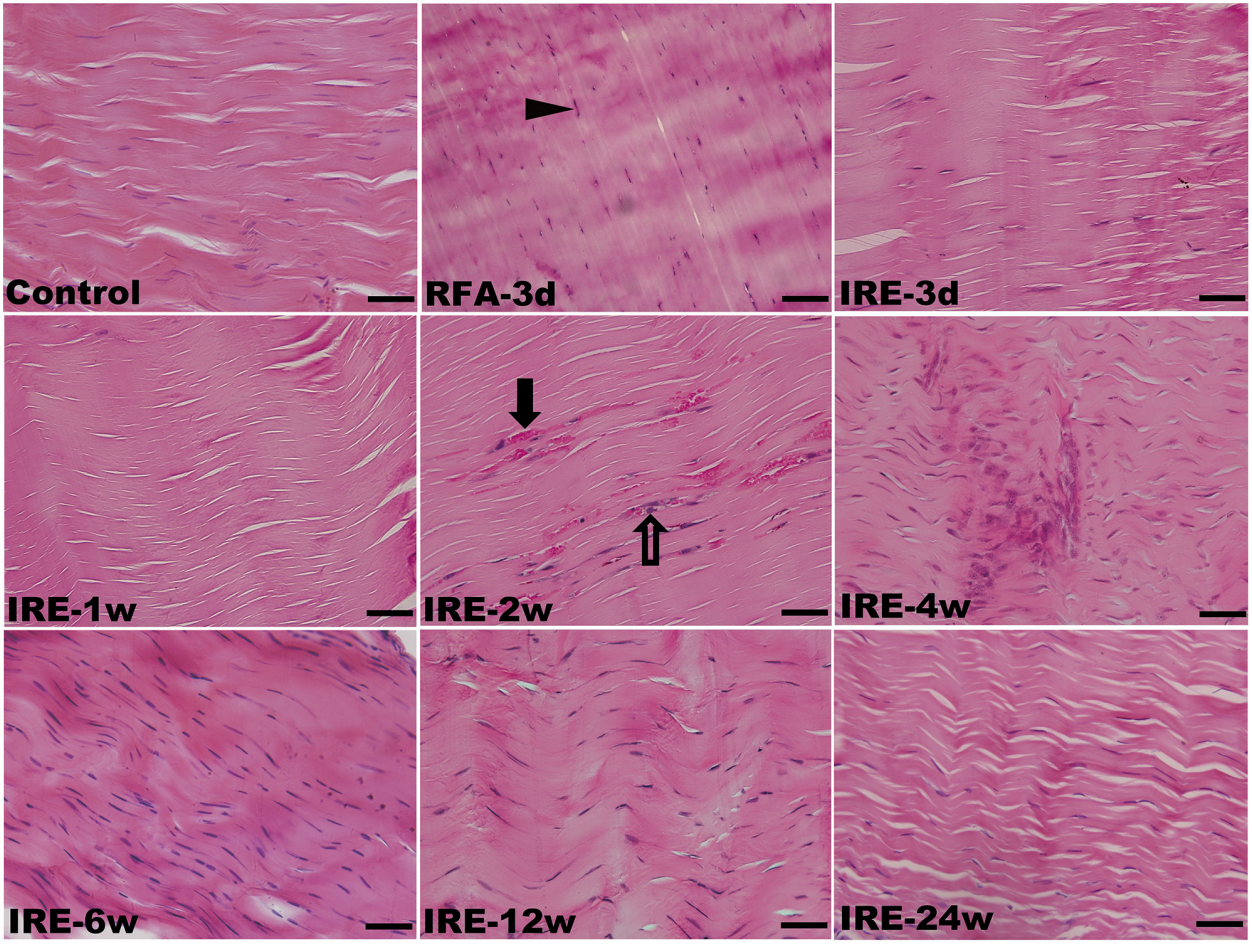
H&E staining of the rabbit ATs following sham operation, RFA and IRE ablation in longitudinal section. (RFA-3 d) Coagulative necrosis induced nuclear pyknosis and karyorrhexis (arrow head); (IRE-1 w) complete IRE ablation and removal of tenocytes; (IRE-2 w) regeneration of blood capillaries (black arrow) and tenoblasts (hollow arrow). The scale bars represent 50 μm.

**Fig 5:**
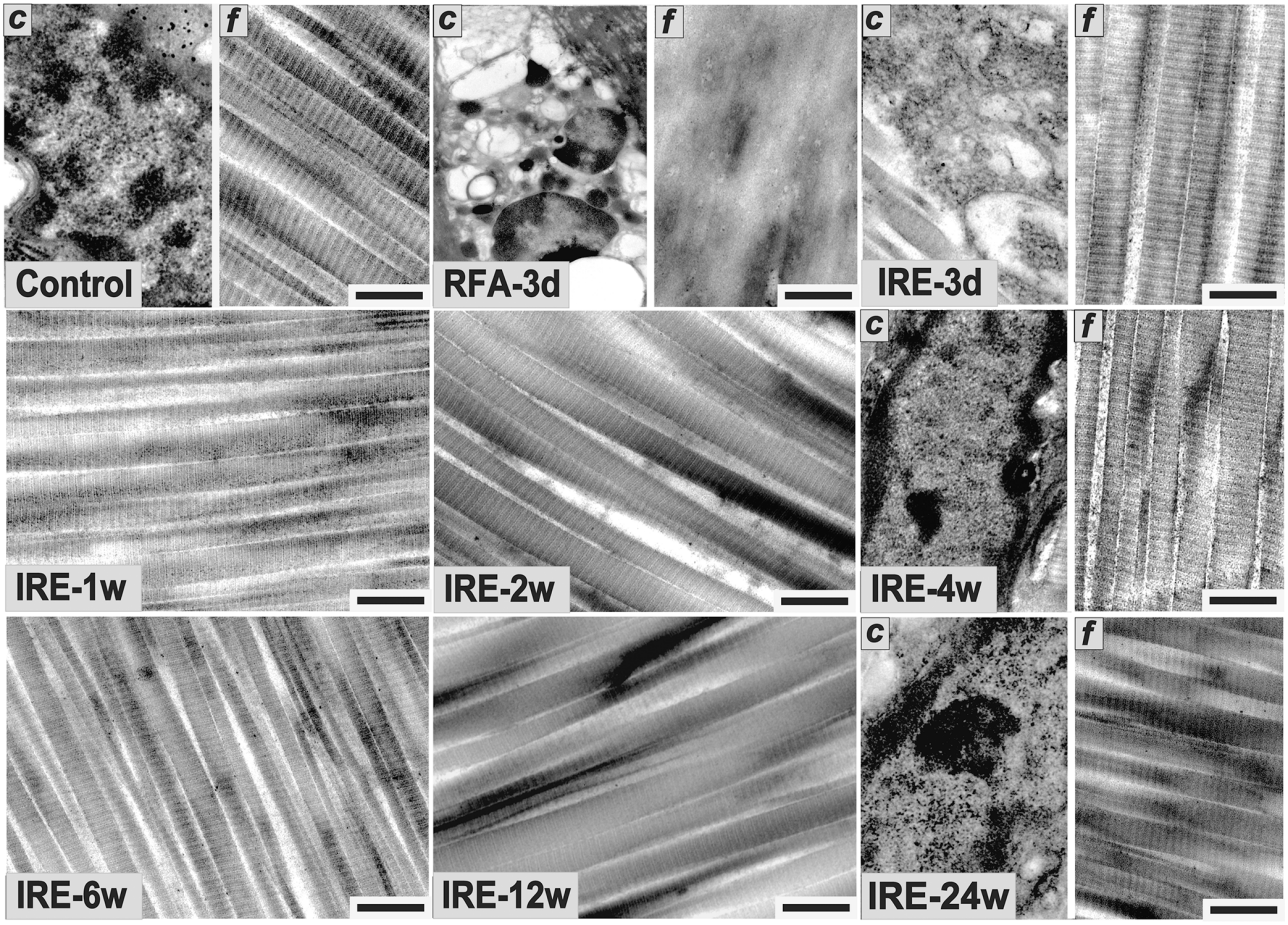
Histological evaluation of the rabbit ATs following sham operation, RFA and IRE ablation with TEM. The local details of tenocytes (*c*) and collagen fibers (*f*) are separated by a dividing line in the same photograph. (Control) A normal tenocyte and obvious transverse bands on each parallel collagen fibril with small interfibrillar gaps. (RFA-3 d) A typical necrotic cell with a dissolved membrane and blurred cytoplasmic components. Collagen fibrils were difficult to distinguish from the degenerative ECM. (IRE-3 d) A necrotic tenocyte with a blurred cell membrane, degenerative organelles and a dissolved nuclear membrane. The architecture of the collagen fibrils is relatively intact. (IRE-4 w) A regenerated tenoblast with a clear nuclear membrane and nucleolus. Collagen fibrils were slightly separated by small interfibrillar gaps. (IRE-6 w) Larger interfibrillar gaps and smaller fibril diameters. (IRE-24 w) An approximately normal tenocyte with a clear nuclear membrane and conspicuous nucleolus. Collagen fibrils appeared normal with clear bands and small interfibrillar gaps. The scale bars represent 0.5 μm.

**Fig 6:**
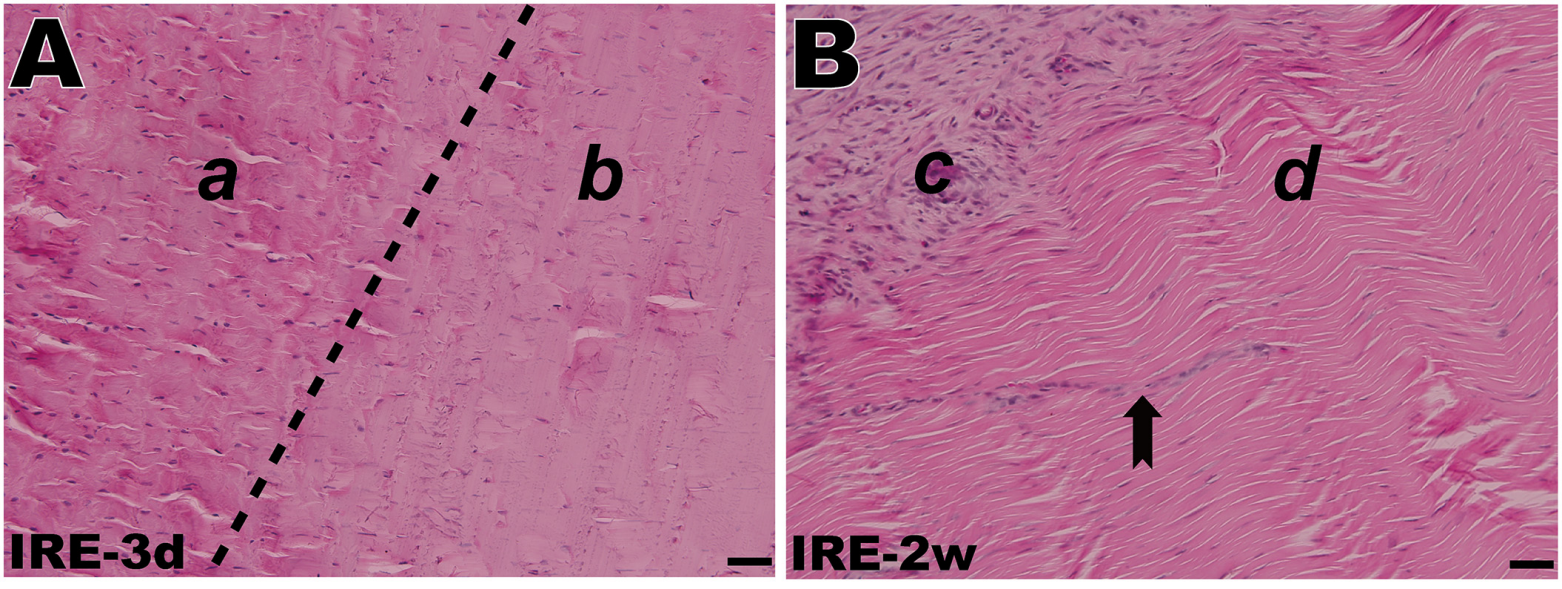
H&E staining of the rabbit AT after IRE ablation. (A) A well-demarcated margin between the non-ablated region (*a*) and the ablated region (*b*) 3 days post-IRE. (B) Peripheral paratenon formation and endotenon ingrowth 2 weeks post-IRE. Tenoblasts migrating from the periphery (*c*) towards the interior (*d*) in alignment with the collagen fibers (dovetail arrow). The scale bars represent 50 μm.

**Fig 7:**
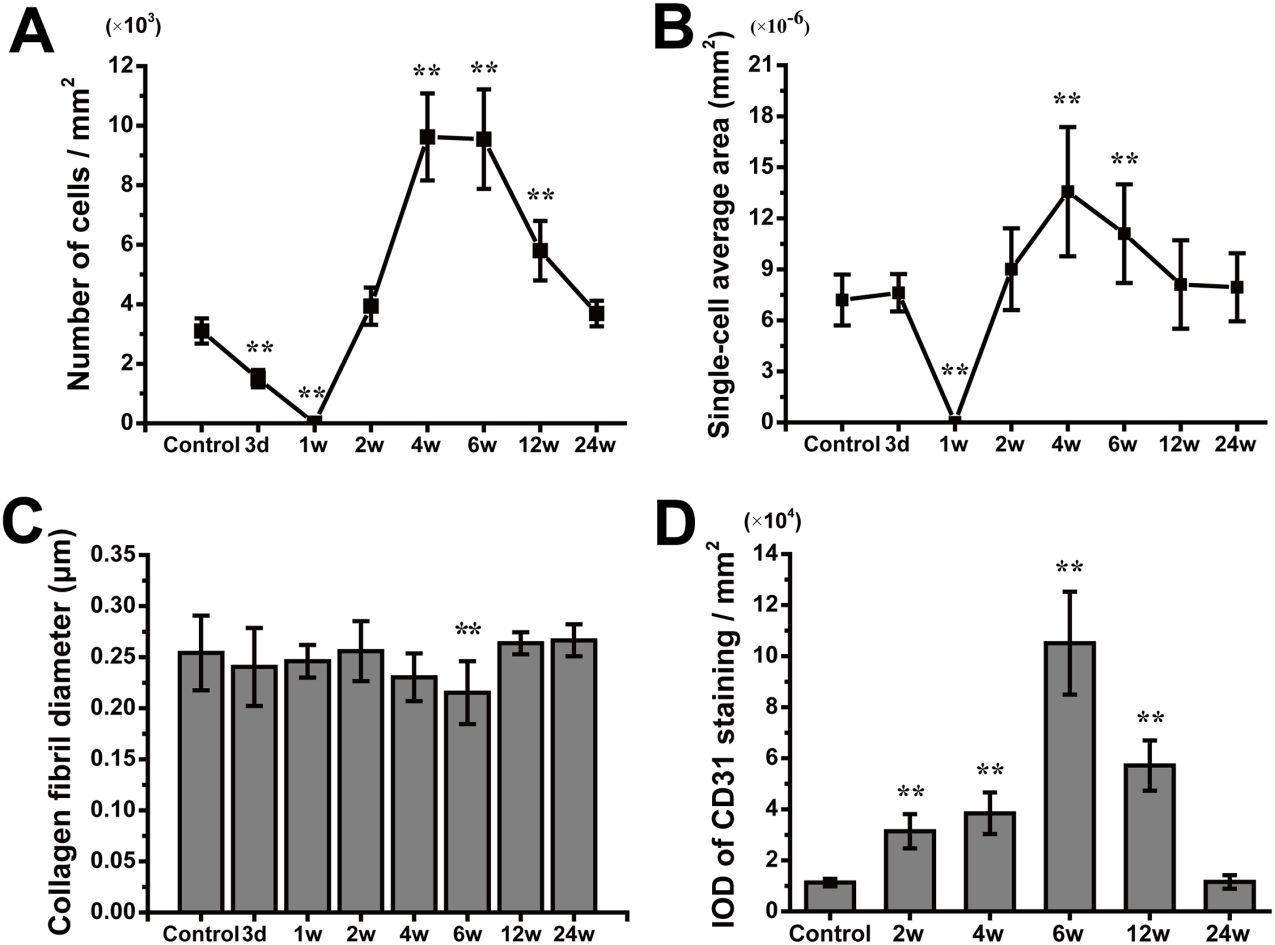
Quantitative analyses of the histological observations. Quantification of the number of cells per square millimeter (A), the single-cell average area (B), the average collagen fibril diameter (C) and the IOD of CD31 staining per square millimeter (D) in the control and IRE groups at each time point. **P < 0.01 vs. controls, two-way ANOVA with Bonferroni multiple comparison post-tests. The error bars represent the standard deviations.

In the control group, H&E staining revealed that the collagen fibers were wavy but parallel to the major axis of the tendon. Sparse tenocytes with flat nuclei were distributed in the interspaces of dense fibers (Fig 4). TEM revealed a transverse band on each parallel collagen fibril and narrow interfibrillar crannies. However, in the RFA group, disintegration, liquefaction and coagulative necrosis with nuclear pyknosis and karyorrhexis were evident 3 d after ablation. Collagen fibrils were difficult to distinguish from the tangled mass of degenerative extracellular matrix (ECM). However, the architecture of collagen fibrils appeared to have remained intact during the initial 2 w after IRE (Fig 5). In the IRE group, the cell structure apparently disappeared 3 d after ablation, leaving a large number of condensed and abnormal nuclei. There was a well-demarcated margin between the ablated and non-ablated regions (Fig 6). The combination of 1800 V/cm and 90 pulses was sufficient to ablate all cells within the target region 1 week post-IRE (Figs 4 and 7A). The IRE-2 w specimen was characterized by the regeneration of tenoblasts and blood capillaries. Several round or oval-shaped tenoblasts were observed migrating from the peripheral non-ablated region, as well as from the ingrowing endotenon towards the intermediate region. In the IRE-6 w specimens, the collagen fibrils were slightly separated by small interfibrillar gaps, and their average diameter had decreased (Figs 5 and 7C). The number of cells per square millimeter and the individual cell area were both at high levels from 4 w to 6w (Fig 7A and 7B). Modeling and maturation of the AT were evident in IRE group between 12 w and 24 w. Over time, collagen realignment and cellular distribution gradually returned to control levels.

### Immunohistochemistry and Angiogenesis Analysis

**Fig 8:**
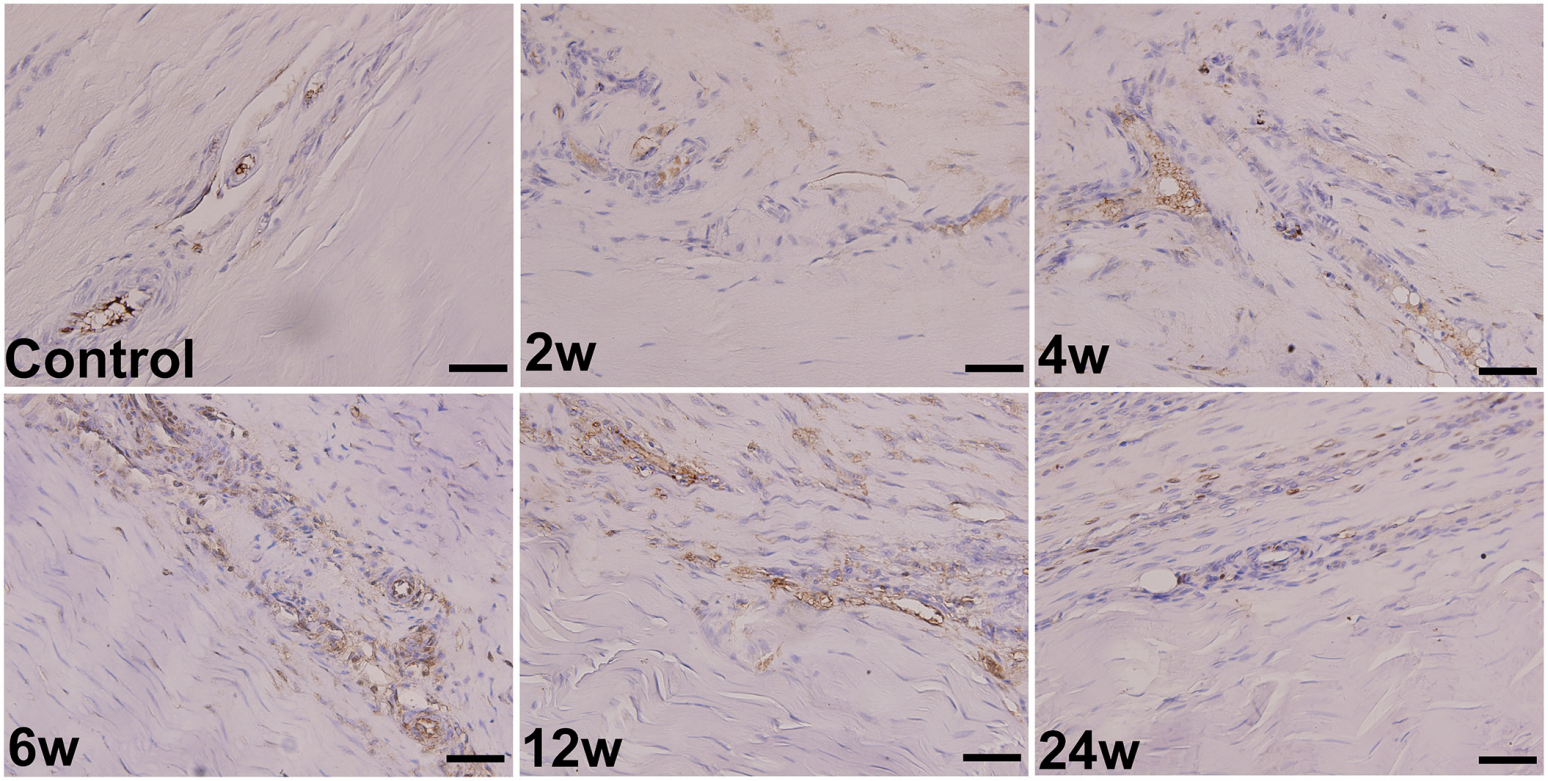
Immunohistochemistry. Immunostaining of endothelial cells with an anti-rabbit CD31 antibody on tissues of normal AT (control) and IRE-treated ATs (from 2 w to 24 w). The scale bars represent 50 μm.

**Fig 9:**
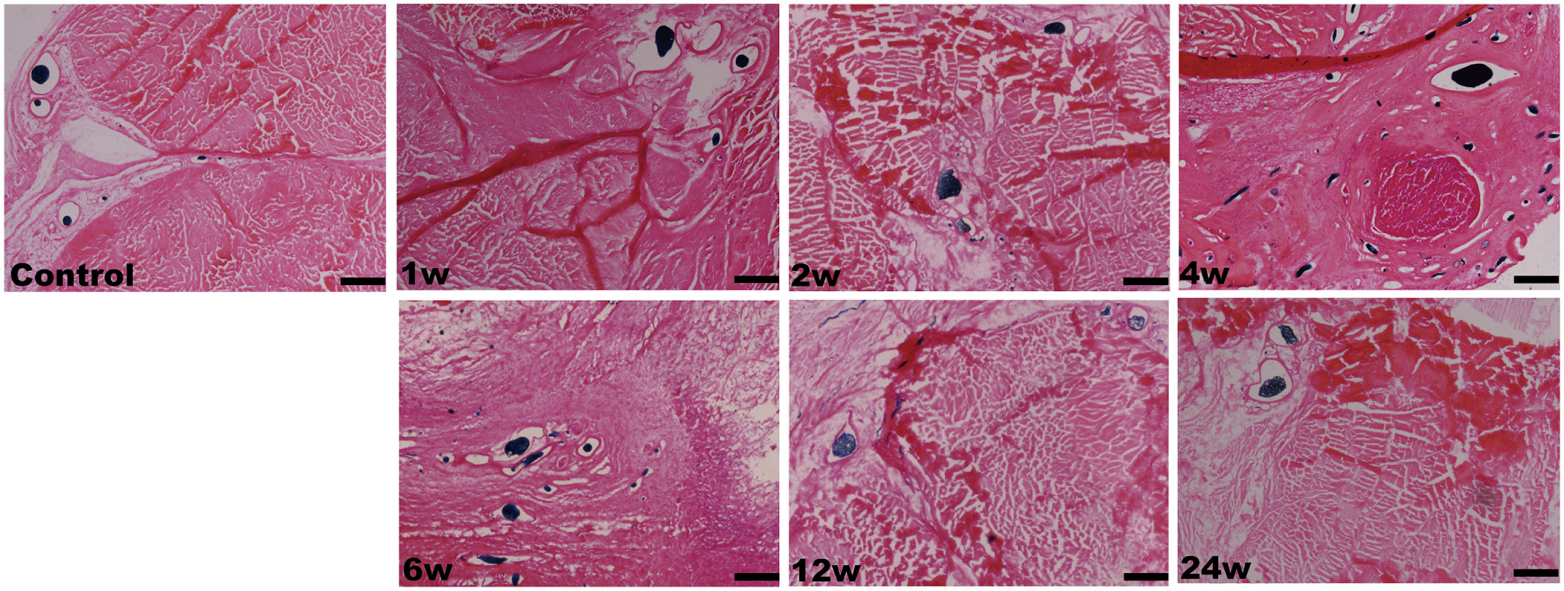
Vascular perfusions. Microfil perfusions of the specimens in the sham operation group (control) and the IRE groups (from 1 w to 24 w) at each time point. The scale bars represent 250 μm.

**Fig 10:**
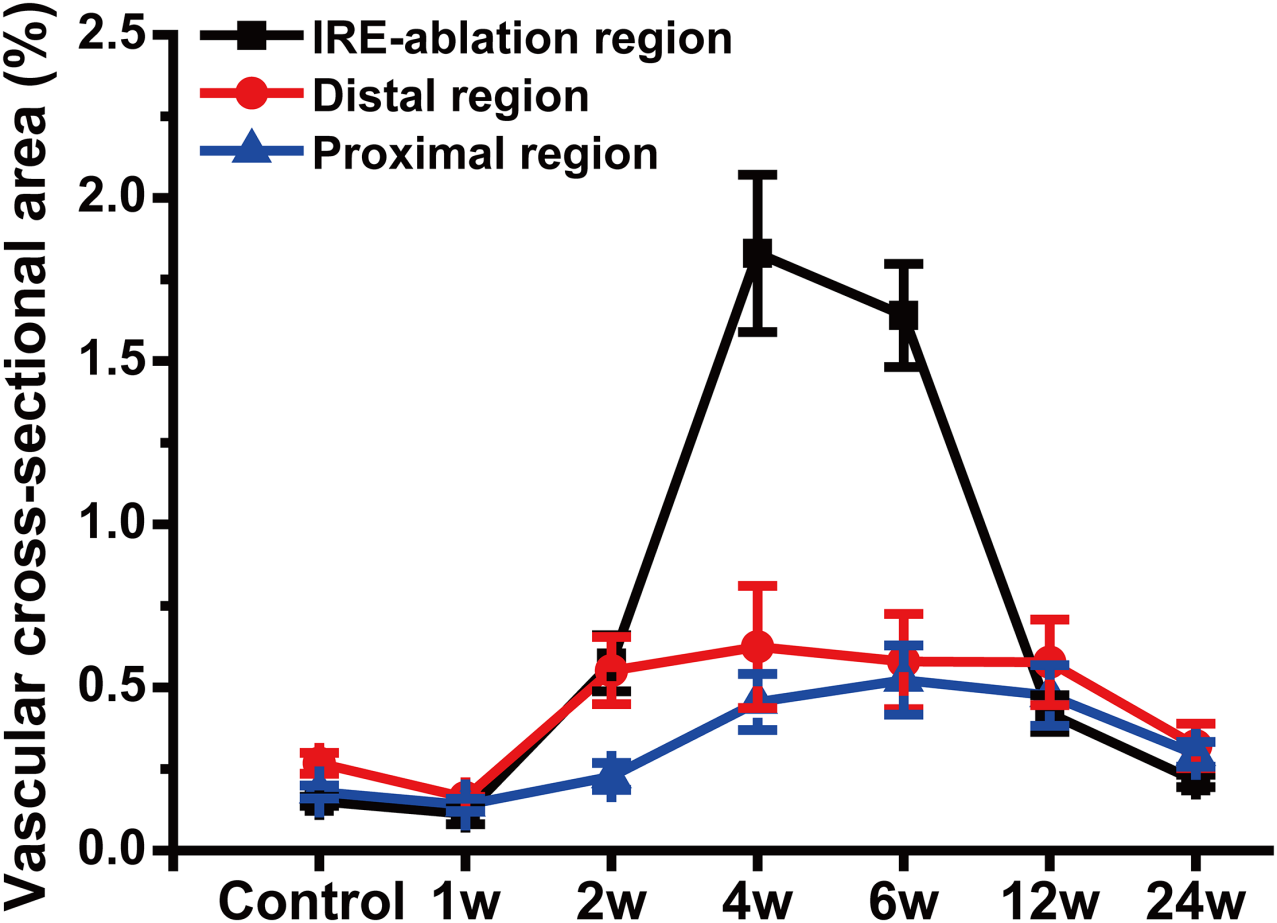
Angiogenesis Analysis. Quantification of the average vascular cross-sectional area in IRE-ablated, distal and proximal regions following sham operations (controls) and IRE ablations. The error bars represent the standard deviation.

We performed this study on the IRE group only. The IOD values of CD31 staining kept higher from 2 w to 12 w than the values observed in controls and peaked at 6 w (Figs 7D and 8). The vascular density decreased at 1 w in the IRE-treated region (Figs 9 and 10), remained at elevated levels from 4 w to 6 w, and ultimately returned to the control levels at 24 w.

### Biomechanical Testing

Table 1 summarizes the biomechanical properties of tendons at various time points after IRE ablation. No significant differences in the length of the IRE-ablated segments were observed. The cross-sectional areas of the AT from 1 w to 6 w were larger than those observed in the control group. The maximum loads at 12 w and 24 w were also significantly elevated (11% and 21%) compared to the corresponding control loads (*P <* 0.05 each). Among all groups, no significant differences were observed in deformation at failure or in stiffness. Most parameters ultimately returned to, or even went beyond, the normal levels.

**Table 1:** The general and mechanical properties of the IRE-ablated AT.

	Time							
	0	3 d	1 w	2 w	4 w	6 w	12 w	24 w
**Cross-sectional area (mm** ^**2**^ **)**								
**IRE-ablation**	7.9(1.2)	8.7(1.6)	9.3(1.2)*	9.4(1.2)*	10.6(2.1)*	10.8(2.4)*	9.1(1.8)	8.1(1.4)
**Control**	7.8(1.1)	7.8(1.3)	7.6(0.8)	7.5(1.0)	7.6(1.9)	7.8(1.3)	8.0(1.4)	7.9(1.3)
**Length (mm)**								
**IRE-ablation**	19.5(2.7)	18.7(3.2)	19.0(2.1)	19.7(3.0)	18.7(4.2)	18.9(2.9)	19.9(4.0)	19.8(3.2)
**Control**	20.0(2.5)	19.2(2.6)	19.9(2.0)	20.2(2.9)	19.4(2.6)	20.0(2.4)	19.8(3.7)	19.5(2.0)
**Maximum load (N)**								
**IRE-ablation**	327(32)	329(27)	315(59)	311(32)	331(76)	325(48)	367(12)*	417(37)*
**Control**	336(48)	341(52)	330(49)	336(42)	329(40)	319(41)	330(26)	330(54)
**Deformation at failure (mm)**								
**IRE-ablation**	5.9(1.2)	5.8(1.0)	6.0(1.3)	6.2(1.6)	6.4(1.6)	6.2(1.6)	6.3(1.6)	6.5(1.2)
**Control**	6.0(1.5)	6.2(1.3)	6.1(1.5)	6.6(1.7)	6.4(1.2)	6.8(1.9)	5.9(1.2)	6.0(1.3)
**Stiffness (N/mm)**								
**IRE-ablation**	105.1(18.6)	100.6(17.3)	116.0(21.4)	100.3(19.6)	100.3(18.5)	98.7(17.2)	103.7(19.8)	106.0(23.0)
**Control**	104.8(17.5)	107.8(15.9)	101.1(19.0)	108.6(17.4)	107.2(19.8)	113.7(15.9)	112.5(21.2)	107.3(16.0)

Mean (SD): *n* = 5 at each time point.

*Significantly different at the corresponding period (*P < 0.05 vs. control, two-way ANOVA with Bonferroni multiple comparison post-tests).

## Discussion

A typical IRE electrical pulse protocol was applied to ATs to induce tenocyte ablation in the target region [10, 15, 16]. Our experiment revealed that IRE ablation spared the structural integrity of the AT without damaging its relevant biomechanical properties.

Tendons are composed of tenoblasts and tenocytes laying within a network of ECM. Tenocytes synthesize collagen and all of the components of the ECM [5]. At the molecular level, the mechanical properties of tendons may be correlated with those of its collagen fibers [17]. In previous animal studies, the mechanical strength of graft tendon was reported to be at its weakest level at 6–8 weeks [18–20]. The mechanical properties of graft tendons/ligaments appeared to be unstable even 12 weeks after surgery [21, 22]. When hamstring tendon grafts were used to perform anterior cruciate ligament (ACL) reconstructions, the full restoration of the biomechanical properties of the intact ACL was not achieved in either in vivo human or animal studies [23]. However, the biomechanical testing results of this study reveal that the maximum load and stiffness fail to exhibit any weakening throughout the experiment. The most significant clinical/biomechanical observations of differences between the two methods may be the fact that all rabbits in the IRE group could fully bear weight without a bearing brace and no ruptures occurred during the experiment. In contrast, rabbits in the RFA group had AT rupture simply by supporting their own weight. IRE preserves the collagen and all components of the ECM. A loss of the cellular component did not immediately affect the mechanical properties of the ligament. Collagen synthesis ceased during the early postoperative days but recovered quickly with the regeneration of tenoblasts. The collagen content increased over time and became even higher than that observed in controls, most likely because of the increased cell density and the increase in collagen expression during the regeneration process. This rapid recovery may be closely correlated to the decreased injury of collagen fibers and the increased levels of collagen synthesis that are accompanied by revascularization and the recovery of cellularity.

The target tendon in this study was included within the zone of hypovascularity [24]. Although the local edema observed in the ECM at 1 w has the potential to cause ischemia in the IRE-ablated segment and to induce a corresponding reduction of blood flow to the other two untreated segments, a complete recovery of the blood supply was subsequently observed. The tendons/ligaments after IRE underwent several phases of characteristic changes that are similar to the course exhibited by the graft tendon, including a phase of hypocellularity and no detectable revascularization, followed by phases of proliferation, revascularization, and ligamentization. Compared to graft implantations, tendons do not completely devascularize after IRE ablation. Rather, they were restored to at least normal levels at 2 w, indicating an accelerated revascularization. A rapid increase in microvessel density could result from at least two factors: (1) preserving the larger diameter blood vessels can make the recovery of blood supply possible, and (2) sparing the ECM provides the necessary scaffold for revascularization. We hypothesize that a sufficient blood supply contributes significantly to the rapid restoration of cellularity, thereby facilitating tendon rehabilitation over a short period of time. The ECM was spared after IRE, which helps promote the regeneration of tenocytes within the ablated region. It has been speculated that IRE-ablated tendons may heal both extrinsically via the proliferation of tenocytes from the peritendon and endotenon, as well as intrinsically via the migration of tenoblasts from the surrounding non-ablated region and preserved blood vessels [25, 26].

Currently, the most commonly used thermal techniques are RFA and microwave ablation, both of which are modalities based on high temperature. RFA and microwave ablation can cause severe collateral damage by destroying the integrity of the ECM [10]. These side effects often prevent the application of thermotherapy to tumors that are adjacent to tendons or ligaments. We failed to find any in vivo studies evaluating the effects of RFA or microwave ablation on the biomechanical properties or the healing process of tendons. Several studies have reported radiofrequency shrinkage, but the low level of energy used in those studies was insufficient to completely kill the cells [27–29]. In response to the high radiofrequency energy that we applied, tendon rupture inevitably occurred. Although surgical treatments may be used to repair or replace damaged tendons with autografts, allografts, xenografts, or prosthetic devices, the clinical outcomes remain unsatisfactory due to the limited long-term functional recovery that is observed [30, 31]. Successful allograft incorporation could take 18 months to 3 years or more [32, 33]. Similarly, slow autograft maturation can result in graft failure or elongation during the postoperative rehabilitation period for reasons that remain unknown [34]. In previous animal studies examining rabbits, dogs and sheep, new tenoblasts appeared in the allografts within 3 to 4 weeks when a graft tendon was used to repair and reconstruct the defect of a ligament, such as the ACL [35, 36]. A significant increase in the number of blood vessels often occurs at 4 weeks for autografts and at 12 weeks for allografts [25, 37]. Newly formed vessels have been observed to progress from the periphery of the graft to span the entire graft diameter at 12 weeks [38, 39]. However, none of these studies demonstrated that the microvascular volume exceeded the normal control levels.

The finding that IRE can be used to ablate substantial volumes of tissue in various organs has been confirmed in animal models and in humans with lung, prostate, kidney, and liver cancers [40, 41]. IRE is able to accurately ablate tumors while giving nearby tendons an opportunity to completely regenerate and functionally recover. Based on these advantages, this technique may help protect the structural integrity of tendon/ligament tissue when treating bone tumors that are adjacent to critical tendons/ligaments.

Several limitations of this study must be acknowledged. First, this was a short term set of experiments with a limited number of experimental animals. As a result, our findings may not adequately represent the realities of the entire population, and additional studies are warranted. Second, the healing process after the IRE ablation of a wide range or a long segment of tendon or ligament may not match that of a target tendon with a limited length. Finally, this study was also limited by its focus on tendons because although the molecular composition of tendons, joint capsules and ligaments is similar with respect to collagen fibers, the number and arrangement of inter-fibrous bonds may vary among these different tissues.

When considered as a whole, the pathological data now available indicate that the advantages of IRE over thermal ablation technologies (such as RFA) are significant in tendons. Improved procedural planning, monitoring, and lesion resolution could have a significant impact when performing ablations of tumors near vital tendons or ligaments.

In conclusion, IRE is an effective means of performing normal tendon ablations. Because of the observed advantages of IRE, such as the preservation of collagen fibers and blood vessels, the possibility of rapid revascularization in the ablated area, and the early creeping substitution of normal tenocytes, the IRE ablation method for treating musculoskeletal tumors may be important in maintaining the integrity and function of remaining tissues.
